# Dementia care practices among community healthcare workers in Vietnam: a qualitative descriptive study

**DOI:** 10.1186/s12877-023-04199-0

**Published:** 2023-09-14

**Authors:** Hong Le Huynh-Truong, Yea-Ing Lotus Shyu, Thuy Khanh Linh Tran, Hsiu-Li Huang, Viet Anh Do

**Affiliations:** 1 Graduate Institute of Clinical Medical Sciences, College of Medicine, Chang Gung University, Taoyuan City, Taiwan; 2 School of Nursing, Faculty of Nursing-Medical Technology, University of Medicine and Pharmacy at Ho Chi Minh City, Ho Chi Minh City, Vietnam; 3 School of Nursing, College of Medicine, and Healthy Aging Research Center, Chang Gung University, Taoyuan City, Taiwan; 4 Dementia Center, Department of Neurology, Linkou Chang Gung Memorial Hospital, Taoyuan, Taiwan; 5 Department of Nursing, Kaohsiung Chang Gung Memorial Hospital, Kaohsiung, Taiwan; 6 Department of Gerontological Care and Management, Chang Gung University of Science and Technology, Taoyuan, Taiwan; 7 School of Nursing, Chang Gung University, 259 Wenhua 1St Road, 33302 Taoyuan, Taiwan; 8 Department of Long-Term Care, National Taipei University of Nursing and Health Sciences, Taipei City, Taiwan; 9 Head of Home Community Care, Royal Freemasons’ Benevolent Institution, Suite 2, Level 12, 2 Park St, NSW 2000 Sydney, Australia

**Keywords:** Community healthcare professional, Confidence, Dementia care practices, Dementia knowledge, Older persons with dementia

## Abstract

**Background:**

Vietnam is one of the most rapidly aging countries in the world and the likelihood that someone may have dementia rises dramatically as the population ages. Although caring for persons living with dementia is important, little is known about the circumstances under which community healthcare professionals in Vietnam provide dementia care. This study aimed to describe the practice of caring for persons with dementia among community healthcare professionals in Vietnam.

**Methods:**

This qualitative descriptive study was conducted with 23 community healthcare professionals recruited from 10 primary healthcare centers, representing 10 of 24 districts in Ho Chi Minh City, Vietnam. Participants were physicians (*n* = 11), physician’s assistants (*n* = 8) and community nurses (*n* = 4). Data were collected through in-depth face-to-face semi-structured interviews. Interview data were audio recorded, transcribed verbatim, and analyzed using content analysis.

**Results:**

The mean age of the 23 participants was 44.6 ± 8.8 years; most were female (*n* = 16, 69.6%); and the mean time of working in the field of dementia care was 15.9 ± 8.4 years. Analysis of the interview data revealed five categories, which informed how care was provided: 1) Knowledge about dementia and its prevalence among older adults; 2) Identification of dementia in Vietnam; 3) Lack of attention to early diagnosis of dementia and difficulty in providing continuous care; 4) Dependence on family members for prompt and continuous care; and 5) challenges to providing dementia care. Despite having knowledge about dementia, some healthcare professionals incorrectly viewed dementia as an inevitable part of the ageing process. Participants reported that their limited training and practical experience in caring for persons with dementia caused a lack of confidence in dementia care.

**Conclusions:**

The quality of care provided to persons living with dementia was negatively impacted by the limited training of healthcare personnel. The diagnosis, treatment, and provision of supportive services to persons living with dementia and their families are substantial challenges for the Vietnamese healthcare system. It is crucial to initiate and cultivate dementia care education programs aimed at expanding curricula for physicians, physicians’ assistants, and nurses.

## Background

Dementia is a condition that affects older adults and is characterized by a combination of degenerative disorders that impair memory, reasoning, behaviors, and the capacity to carry out activities of daily living (ADLs) [1]. The World Health Organization (WHO) reports that not only is dementia is one of the main causes of disability and dependency among people worldwide, but it is also the seventh cause of death among all diseases [1]. Statistics from Alzheimer’s Disease International (ADI) reported more than 55 million people were living with dementia worldwide in 2020 [2]. This number is estimated to reach 78 million by 2030 and 139 million by 2050, with developing countries accounting for most of the increase [2]. In 2022, ADI estimated that approximately 10 million new cases of dementia are diagnosed each year, or one case per 3.2 people [2]. Globally, it is estimated that 75% of dementia cases are undiagnosed, with lower- and middle-income countries reporting a rate of up to 90% [3].

In industrialized countries, it is recommended that patients schedule a visit with their primary care physician for an evaluation if there is a concern about the possibility of dementia [3]. Primary care physicians and community nurses are the best specialists for providing dementia care by treating, counselling, connecting with, and helping older adults with dementia [3–6]. This is because persons with living with dementia and their families prefer that cognitive screening and dementia services be provided at primary care facilities close to their homes [3, 7]. However, these services are not always available in underdeveloped countries.

According to statistics from The World Bank, the country of Vietnam is experiencing one of the most rapid increases in its population of older adults. In 2019, in a total population of 96.21 million, 11.86% were people over the age of 60 and it is estimated that the proportion of individuals 65 years and older will be 14.2% in 2036 [8, 9]. In Vietnam, healthcare management for older adults is implemented at the district level via community healthcare facilities [10, 11]. Healthcare professionals at community healthcare centers are responsible for early identification and disease management for the older adults in the community who are experiencing chronic illnesses, such as hypertension, diabetes, and dementia. Each center has a psychiatric physician who is in charge of mental healthcare for older adults, which includes dementia care. Other healthcare professionals, including physicians, physician’s assistants, or clinal nurses at each health center also provide care and support for older adults.

Dementia has been recognized as a public health issue since 2017, which is a result of the country's rapidly ageing population [12]. In January of 2022, an integrated national action plan for Non-Communicable Diseases (NCD) and mental health disorders was approved by Vietnam’s Prime Minister via Decision No 155/QD-TTg. This plan involves the goal of developing healthcare guidelines to be attained by 2025, however guidelines for the diagnosis and care of patients with dementia have not yet been developed. Therefore, although community healthcare practitioners in Vietnam provide services to older adults with dementia, these patients have not received a formal diagnosis of dementia and the healthcare providers often have little knowledge or training about dementia care [12].

Studies examining the care currently provided to older adults in Vietnam by community healthcare personnel, particularly those with dementia, are scarce. This lack of research might explain the lack of effective interventions as well as the inadequate updating of healthcare policies and practices of care for this vulnerable population. Therefore, the aim of this study was to describe the current dementia care practices provided by community healthcare professionals in Vietnam. Findings from this study could be used to develop interventions to improve delivery of dementia care, develop interventions for older persons with dementia, and as a guide for updating healthcare policies in Vietnam.

## Methods

### Study design and participants

A cross-sectional descriptive qualitative study was conducted using in-depth face-to-face interviews to understand the variables that inform the practice of dementia care for community healthcare professionals in Vietnam. Professionals were recruited from 10 primary healthcare centers in Ho Chi Minh City, Vietnam, which represents 10 of the 24 districts in the city. Criteria for participation included persons over 20 years of age and having worked as a community healthcare professional for older adults for at least 5 years. Sample size was determined by the theoretical saturation of the data, which was reached when the same patterns of concepts and incidents were obtained without any new properties [13].

### Data collection

Data were collected from September 2017 to March 2018 using a researcher-developed semi-structured interview guide comprised of questions based on literature about dementia care [4] and family caregivers [7, 12], which was reviewed by two experts in dementia and three Vietnamese healthcare providers. The questions were designed to understand participants’ general knowledge of dementia care, as well as their attitudes, confidence and practice of dementia care for older persons with dementia as well as their perceived strengths and challenges in caring for persons living with dementia (Table 1). Prior to beginning the study, two pilot interviews were conducted to ensure the clarity and understandability of the questions. No changes were made to the interview questions. Interviews were audio recorded with the permission of each participant and each interview lasted 30–50 min. The interviewer maintained detailed field notes during each interview to record nonverbal behaviors of the participant. Data collection and analysis were conducted concurrently. Data collection discontinued when categories became saturated, which occurred with the last two interviews. Confirmation of data synthesis and findings was conducted with eight participants in October 2022.

**Table 1:** Semi-structured interview guide for community healthcare professionals

Interview questions
1. What do you know about dementia in general? Specifically, what is your definition and what are the causes? Is there a cure for dementia/Can you prevent dementia?
2. In your opinion, what type of care is needed for older persons living with dementia?
3. As a health care provider, how do you diagnose, treat, and care for older adults with dementia?
4. In your opinion, when a community health care professional suspects a person has developed dementia, how should this information be communicated?
5. What are your perspectives on early diagnosis of dementia?
6. How do you respond in a situation where a community health care professional is unable to diagnose a patient as having dementia?
7. How do you respond in a situation where a community health care professional is unable to continue to provide care for one of your patients living with dementia?
8. Can you provide an example of a patient you diagnosed as having dementia? If yes, can you describe how you diagnosed the patient and what did you do to manage behavioral problems, if they were present?
9. Do you provide caregivers with health education about recognizing and managing symptoms of dementia? If yes, what information do you provide to them?
10. How would you rate your confidence in performing the practices you have described?
11. As a health care professional, what do you do to identify older patients you suspect of having dementia?
12. Are you familiar with any of the available tools for assessing cognition? If yes, do you use any of these assessment tools for you older patients? How frequently to you use an assessment tool? Have you experienced any difficulties using the tool?
13. Have you had experience caring for older adults with dementia and their family members? If yes, please describe strategies and interventions you found to be effective for assisting in their care
14. Have you experienced any difficulties in providing services or providing care for older patients with dementia? If yes, please explain in detail
15. In your community in Vietnam, are there any activities to screen for dementia among older adults? If yes, would please explain in detail how screening is conducted. Who performs this screening?
16. Please describe problem(s) related to regulations/policies that you have encountered when providing care for older persons with dementia

### Ethical considerations

This study was conducted in compliance with the Declaration of Helsinki and approved by the Board of Ethics in Biomedical Research at University of Medicine and Pharmacy at Ho Chi Minh City (Ethics No 14196-UMP). All participants were provided with a description of the study design and purpose a well as their expected role as a participant. Participants were assured of confidentiality and anonymity of their data as well as being able to request that their data not be used in the analysis. All participants provided a signed informed consent form prior to interviews.

### Data analysis

Interviews were audio recorded and transcribed verbatim in Vietnamese by the researcher as each interview was completed. Transcripts were translated from Vietnamese into English and the translations were validated by two professional English-Vietnamese bilingual transcribers. During data analysis, we used the English translation of the interviews as the primary source. However, we referenced the original transcribed data in Vietnamese to ensure functional and cultural equivalence was maintained. Transcripts of the interview data were analyzed using content analysis with various text analysis strategies [14] using systematic coding and classifying to focus on textual information to determine trends and patterns of words used, frequency and relationships, and structures and discourses of communication [15–17]. These texts were relevant to concepts that inform the practice of dementia care for community healthcare professionals in Vietnam. Each transcript was read line-by-line multiple times to obtain a sense of the overall content. Next, transcripts were read again to identity and select meaning units, which were words, sentences or phrases that conveyed the practice of dementia care in Vietnam. Meaning units were condensed and coded without changing the core meaning of the text. Initial codes were compared across all transcripts and codes with similar meanings were grouped into categories. Subcategories were also identified based on related codes. Three types of codes were used: descriptive, which require little interpretation; interpretative; which is based on the research purpose and the perspectives of participants; and patterns, which are inferential and explanatory, and develop themes or categories. A descriptive code from a participant, who said, “*In Vietnam, there are no dementia screening clinics for the elderly people in the community”,* was coded as “no screening clinics” to summarize the original ideas without further interpretation. An interpretive code from a participant, who said, “*Family members are the main caregivers for patients, healthcare staff only provide supportive care”,* was coded as “family caregivers as the main providers” to reflect the role of the family member. A comment stating, “*Dependance on family members for prompt and continuous care*”, was coded as a pattern code to summarize the expectations of the healthcare professionals on the role of the family caregivers in taking care of persons living with dementia.

Coding was conducted independently by the two researchers who then discussed and compared their findings to reach a consensus. The initial organization of data required constructing a tentative coding list, which is an inductive process that involves naming segments of transcribed data and defining the categories. Then abstraction (extraction) of the categories condenses the data and ensures that categories do not overlap. The tentative coding list was used to guide the subsequent coding process, which involved sorting of categories into sub‐categories according to similarities and differences; these emergent subcategories were grouped into clusters. These clusters and subcategories were revised several times as the coding process progressed and was reviewed by the research team. Codes and raw data were then compared for similarities and differences. Tentative categories and subcategories were discussed with three experts in gerontological nursing and dementia care until a consensus was reached, which resulted in the researchers arriving at five main categories that described community healthcare workers experience of the practice of dementia care in Vietnam.

### Rigour

Strategies to enhance trustworthiness of the study included credibility, dependability, transferability and confirmability [18]. Credibility was established by member checks with the participants, which involved sharing the transcribed interviews and coded text with 23 participants to confirm the findings and clarify any misunderstandings. Dependability was evaluated by maintaining an audit trail of all interview data, coded text, and field notes, checking the findings with the research team, which included two nurses in gerontology and researchers in dementia care. Confirmability was also supported by the audit trail and member checks. Transferability was enhanced by collecting rich data from community participants with different roles as community healthcare professionals.

## Results

### Participants

A total of 23 community healthcare practitioners were interviewed for this study. Participants were comprised of physicians (P, *n* = 11), physician assistants (PA, *n* = 8) and community nurses (CN, *n* = 4). Most participants were female (*n* = 16, 69.6%); the mean age was 44.6 years (SD = 8.8); and the mean number of years working in their professional field was 15.9 (SD = 8.4). The mean number of older persons with dementia participants had cared for in their facilities or departments was 13.7 (SD = 12.5). Basic demographic information is shown in Table 2.

**Table 2:** Participants’ basic demographic information (*N* = 23)

Characteristic	n (%)	Mean (SD)
Gender		
Female	16 (69.6)	
Male	7 (30.4)	
Age (years)		44.6 (8.8)
Profession		
Physician	11 (47.8)	
Physician assistant	8 (34.8)	
Nurse	4 (17.4)	
Experience working in community healthcare (years)		15.9 (8.4)
Number of older adults with dementia in your care		13.7 (12.5)

*SD* Standard deviation

### Dementia care practices for older adults in Vietnam

Content analysis revealed five categories, which informed community healthcare professionals in Vietnam about their practice of dementia care: knowledge about dementia and its prevalence among older adults; identification of dementia in Vietnam; lack of attention to early diagnosis of dementia and dementia care; dependence on family members for prompt and continuous care; and challenges to providing dementia care. These categories that informed dementia care were influenced by multiple variables involving the healthcare providers experience, perceptions, interactions with family members, and the community healthcare system in Ho Chi Minh City.

#### Knowledge about dementia and its prevalence among older adults

The community healthcare workers were knowledgeable about the definition, attributes, and symptoms of dementia. Many participants pointed out that dementia is commonly diagnosed in older adults. All participants recognized Alzheimer’s disease as a form of dementia and were aware that there is no cure or treatment available for dementia.

Participants described clinical indicators for dementia as short-term and long-term memory loss, forgetfulness and confusion, and highlighted short-term memory loss as one of the most noticeable symptoms. They were aware that short-term memory loss involved good memory for past events but difficulty in recalling recent events. All participants mentioned loss of time awareness, getting lost, and a reduction in the ability to attend to daily life activities as common symptoms of dementia. Participants described typical symptoms of early dementia as including *“...remembering things in the past and forgetting things in the present, forgetting names, even names of relatives”* [PA14]. All three groups of healthcare providers recognized memory loss as one of the most prominent symptoms of dementia. Awareness that the incidence of dementia was also influenced by comorbidities was voiced by a physician who said, “*Dementia often occurs among senior people or people with diabetes, high-blood pressure or a cerebrovascular incident.”* [P3].

Several participants mentioned that symptoms of dementia were frequently seen in older adults. One physician stated, “*Dementia is a decline in cognitive abilities, memory and forgetfulness and these symptoms are commonly seen in the elderly”* [P5]. Another physician said, *“Dementia is a common disease in older people. The number of people with dementia is increasing with the rise in the number of the elderly”* [P2]. One nurse said, *“Dementia is a condition of memory loss and forgetfulness, which frequently happens among elderly people”* [CN20].

All participants were aware that Alzheimer’s disease is “*one of the most common forms of dementia in older people”* [P10] and all knew there was no formal cure or medical treatment for dementia. The diversity of treatments for the symptoms of the disease, including prescription medications, controlling coexisting diseases such as diabetes and hypertension, and engaging in activities that improve cognition and memory, was mentioned by many participants. One nurse stated, *“Dementia treatment is unavailable; key treatment involves increasing circulation and medications to limit the dementia process”* [CN20]. One physician’s assistant said, *“One treatment is to take a supplement to improve brain activity* [PA10]. The physicians provided the most thorough review of treatment options as exemplified by the following description:

*The treatment depends on the severity of the disease. For example, patients with minor dementia should be guided to change their lifestyles, behaviors, and daily diet; reading books and newspapers for maintaining brain activity; daily exercise, physical exercises can help reduce the disease condition. Patients may use supplementary herbal drugs or drugs that improve circulation and enhance activity in the brain.* [P3]*.*

#### Identification of dementia in older adults in Vietnam

The second category that significantly informed how community healthcare professionals cared for persons living with dementia in Vietnam involved the difficulty in identifying persons with the disease. Participants reported that the diagnosis of dementia was difficult to make as a healthcare provider because of the lack of formal dementia screening programs. Therefore, one pathway to a diagnosis of dementia was often through family members rather than care providers. Most frequently, family members noticed their relative was experiencing memory loss or exhibiting behavioral changes. This was described by one physician who said:

*For most of my older patients with dementia, their disease was detected by family members because of changes in behavior, forgetting their actual age, a loss of awareness of time, getting lost, remembering things in the past, and repetition of something multiple times. Family members usually bring the patient to a community healthcare center or neurological clinic for a medical examination.* [P5]

Another physician had a similar experience, saying, *“Dementia can usually be detected by family members. When the family members recognize the patient’s behavior has changed, they take the patient to see a medical doctor”* [P9].

The second pathway mentioned by healthcare providers was detecting symptoms of dementia in older patients during annual medical check-ups or during a clinic visit for symptoms unrelated to dementia. For example, one nurse said, “*In Vietnam, there are no dementia screening clinics for the elderly people in the community. Dementia screening occurs by chance during an annual medical check-up for elderly people at community healthcare centers*” [CN20]. Another nurse confirmed that this was the experience of many providers, saying “*Community health professionals often identify dementia during an annual medical check-up...”* [CN21]. One physician’s assistant described having identified older adults with dementia when they were being seen in the clinic for an illness: *“I often find patients with dementia when they come to the community healthcare center because they are in need of medical care”* [PA16].

Diagnosis of dementia was not a focus of the healthcare professionals. They explained that recognized diagnostic procedures were not used routinely and most acknowledged a lack of training in a formal evaluation of an older person’s cognitive function. Participants knew about the Mini Mental State Examination (MMSE) as an evaluation tool but did not use it. This was described by a physician’s assistant who said, *“We (our group) do not know how to use any tools to assess elderly people with dementia. We often ask the patients or their relatives to assess their cognition by their individual experiences”* [PA12]. One physician reported their practice knew about assessment tools, but they were not utilized, saying, *“We know the tools of cognitive assessment of elderly people with dementia, but do not yet apply them in our practice. We only ask the patient questions to assess it”* [P7]. Another physician said, *“The MMSE test is the cognitive assessment tool that I am aware of, but I don't use it very often because it takes a while; patients and family members find it difficult to wait. Typically, I do clinical assessments”* [P23].

#### Lack of attention to early diagnosis of dementia and difficulty in providing continuous care

Failure of the general population as well as the Vietnamese government to recognize the importance of prioritizing early diagnosis of dementia through community screenings was voiced by most participants. One physician had the following explanation for the lack of attention, saying, *“The people in Vietnam are less concerned about dementia”* [P9]. Another physician said, *“*(The Vietnamese government) *is not so active in providing healthcare for people with dementia at the local level due to inadequate instruction from the local government”* [P4]. One nurse said, *“Patients with dementia in the community are still under cared for and undertreated”* [CN21]. One physician’s assistant said, *“Healthcare staffs are not really concerned about dementia care”* [PA12].

Community healthcare workers admitted that they felt regret that they could neither identify dementia earlier nor introduce patients to specialized hospitals or facilities for obtaining an accurate diagnosis. One physician’s assistant shared the following: *“I regret that I could not identify dementia earlier. I also feel disappointed because of my limited contribution to dementia care for my patient”* [PA10]. One nurse said, *“I found that I lack abilities to care for my patients”* [CN21]. One physician said, *“If community healthcare professionals cannot identify people with dementia, the patient should be introduced to a specialized hospital for an accurate diagnosis”* [P9].

There was a consensus among physicians, physician’s assistants and nurses that an early diagnosis of dementia was important because treatments could be initiated that might delay the progression of dementia, which would reduce the caregiver burden for family members. One physician said, *“Early diagnosis of dementia is very important. Early diagnosis and timely treatment can slow down the disease process”* [P4]. One physician’s assistant stated, *“Early diagnosis of dementia would reduce the care burden of the family and society because early diagnosis and early intervention of dementia will slow the progression of dementia”* [PA13].

Community healthcare workers also mentioned that it was difficult to provide continuity for patients with dementia. One nurse said, *“Community healthcare professionals have to monitor and continue caring for a patient with dementia. In situations where a community healthcare professional is unable to provide continuous care, family members are asked to take the patient to another healthcare facility that will provide continuous treatment and care, which will be paid for by health insurance”* [CN20]. One physician’s assistant lamented not being able to continue caring for the patient and needed to *“hand over* (the patient) *to other healthcare professionals”* [PA17]. One physician stated, *“When community healthcare workers are caring for a patient with dementia, they are not allowed to quit. In a situation where we are unable to continue caring for a patient with dementia, we try to obtain assistance and support from social organizations or the patient’s community”* [P10].

#### Dependance on family members for prompt and continuous care

Participants believed the design of the healthcare system in Vietnam made it difficult to provide the prompt and continuous care critical for persons with dementia. Participants mentioned that it was the family caregivers who were the main providers of dementia care, and that more effort should be made to offer caregivers education about dementia, activities that slow memory loss, helping patients adhere to treatment for comorbidities, spiritual support, training in dementia care and management of dementia and related behavior problems, such as getting lost.

One physician said, *“Family members are the main care-giver for patients, healthcare staff only provide supportive care”* [P11]. A physician’s assistant recommended the following: “*Family members should pay more attention to the patient. Reducing memory impairment by frequently talking, sharing, going to entertaining places, and encouraging relationships with other elderly people”* [PA12]. A physician’s assistant said, *“Guide relatives to help the patient do memory exercises such as book reading. And be sure that they follow treatment plans accompanying a physical illness such as hypertension, diabetes”* [PA19]. One nurse made the following suggestion, saying, *“Spiritual care for patients is necessary. Family members should also be encouraged to talk frequently with the person with dementia. Patients forget. For instance when a patient forgets their child’s name, they can help to improve their memory by repeating their child’s name”* [CN20].

Providing dementia care training for family members as a primary intervention by encouraging them to take an active role in fostering as much independence as possible was suggested by almost all participants. One physician’s assistant said, *“Educate their family members how to continue caring for the client “*[PA16]. A physician recommended: *“The most effective intervention is the care from relatives. Educate relatives how to care for and monitor the patient at home, such as encouraging the patient to do everyday activities by themselves”* [P6]. Another physician said, *“Guiding family members, letting the patient be involved in social activities, watch television, and go out with friends”* [P9].

Participants had several suggestions for management techniques. Forgetting to eat is a typical symptom of dementia and one that participants thought family members could address. A physician stated, *“If a patient forgets to eat, someone needs to remind the patient to eat on time”* [P10]. A physician’s assistant recommended the following: *“Instruct family members to establish a schedule to check on the patient's meals”* [PA15]. Getting lost is also common for persons with dementia. One nurse suggested the following as an intervention for patients who become lost or feel disoriented to places, saying, “*The relatives should make sure they have identification* (when they go out), *which should include the patient's name, address, and phone number so that in the event that the patient goes missing, someone can call the family*” [CN20]. “*Don't allow the patient go out alone*”, advised by one physician’s assistant [PA19].

### Challenges to providing dementia care

The lack of dementia-related legislation, community resources, and professional training were among the challenges to dementia care practices described by participants. Most community healthcare professionals in our study reported feeling less confident in their ability and capacity to provide dementia care because they did not have enough of the necessary training. When asked to rate confidence in caring for patients with dementia, one physician stated, “*I am not confident because I have not had much contact with patients with dementia and I haven’t been trained in providing dementia care*” [P2]. Another physician said, “*I still feel apprehensive when meeting with patients with dementia since community health workers are not skilled in dealing with this disease*” [P23]. One nurse said, “*I am not confident because I lack experience, need more training about dementia*” [CN17]. A physician’s assistant reported, “*I am not confident because I have not had many patients with dementia. I have little experience and not enough annual training in dementia care*” [PA19].

Vietnam has a program aimed at caring for older adults as well as a general mental healthcare program, which includes components for older adults with dementia. However, However, at the time of these interviews, the health policy guidelines and action plans had not yet been established for care of older adults with dementia in Vietnam. This was a significant challenge for participants. One nurse stated, “*Currently, there are no regulations or policies for dementia care of the elderly at the primary care level. Caring for patients with dementia is not integrated into the general healthcare program for elderly people* [CN21]. One physician’s assistant said, “*The government has not developed any particular health policies for elderly people with dementia. The care program for the elderly with dementia should be a component of care for the elderly in general”* [PA10]. One physician said, *“Currently, there are no care programs for the elderly with dementia integrated into the mental healthcare programs. Thus, there are many elderly people with dementia who have not been diagnosed and are not cared for”* [P15]. Another physician said, “*The Ministry of Health has not issued any regulations on the management, treatment, or care of the elderly with dementia in the community”* [P23].

An additional challenge mentioned by participants was the high cost of medications. The recommended medications are very expensive and not fully covered by Vietnam’s general health insurance. Therefore, patients and their family members struggle with treatment costs, which also included consultations with specialists, dementia caregiver expenses, and pharmacological therapies. One physician said *“The list of medications to treat dementia that is paid by health insurance is limited. The more effective medication treatments are too expensive for the patients to buy. Patients’ financial condition plays a major role in dementia care”* [P5]. One nurse said, *“The type of healthcare service for dementia that can be offered depend on the patient’s financial status.”* [CN20].

## Discussion

To the best of our knowledge, this is the first qualitative study to explore dementia care practice for older adults among community healthcare professionals in Vietnam. Participants in our study were knowledgeable about most aspects of the disease of dementia, including symptoms of dementia and an awareness that dementia is not a curable disease. Most healthcare professionals reported they had not been the one to identify their patients as having dementia because there is no routine dementia screen process in Vietnam. Rather, dementia was apparent when an older relative underwent an annual medical check-up, sought needed medical care, or a family members recognized their relative was exhibiting dementia-related symptoms, such as memory loss or behavior changes. The design of the Vietnamese healthcare system made providing the ongoing care necessary for persons with dementia difficult. Thus, participants considered family members one of the most important components of providing dementia care.

Several participants mentioned that the diagnosis of dementia most frequently occurs in older adults. Although dementia is not a characteristic of ageing [1, 3], a recent survey found more than 62% of medical professionals globally believe that dementia is a natural part of ageing [19]. Our finding that inadequate knowledge about dementia was barrier to recognizing dementia’s early symptoms, diagnosing the disease, and providing care is similar to studies conducted in rural settings of China and Europe [20–22]. In addition, the limited training and experience with dementia was reported to be one of the most important barriers to providing dementia care as a community healthcare professional. These findings echo those of studies conducted with community care providers in areas of China with undeveloped dementia care programs [7, 23].

Nearly all participants reported that their patients often received a diagnosis of dementia by chance during an annual health screening. This was typical because dementia care is not a public health priority in Vietnam and there is no formal dementia screening program [24]. Whereas most countries encourage individuals to visit their primary care physician as a first step towards a diagnosis of dementia [3], it is not unusual for low- and middle-income countries to not conduct dementia screening [8, 25, 26] where it is estimated that over 75% of people living with dementia are undiagnosed [2]. The fact that only 20% to 50% of primary care patients are identified as having dementia in high-income countries [8, 20] suggests the gap in providing a formal diagnosis is larger in low- and middle-income countries. These results emphasize the need for dementia screening programs in Vietnam, which that could deliver early identification of dementia among the general population.

Participants reported that family members served as the primary caregivers for their patients with dementia, which is supported by a previous studies reporting that, regardless of their illness, 93.1% of older adults in Vietnam are cared for by family members [8, 26]. This is because most family members do not want a relative living in nursing care facilities, which are uncommon in Vietnam [27]. The high proportion of family caregivers is the result of the Chinese cultural tradition of filial piety, which remains strong in Vietnam [28, 29]. Filial piety and collectivism are key principles in Chinese cultures, which requires older adults to be cared for by family members. Our findings are similar to those reported for other low- and middle-income countries, where persons with dementia are primarily cared for by family members or other unpaid carers [30]. Vietnam is typical of Asian countries such as China and Korea, where the concept of filial piety may deter families from placing an older relative in a long-term care facility [25, 31]. These findings highlight the need to create and develop suitable support services for family members caring for older persons with dementia, which could be incorporated into home-based care services.

Among the challenges to providing dementia care faced by the community healthcare professionals, the greatest was a lack of confidence in their caregiving abilities. Participants reported that their lack of confidence stemmed from inadequate training and little contact with patients with dementia. These feelings are similar to those expressed by physicians in China who also lacked the skills necessary to treat patients with dementia and their family caregivers [3, 20, 21] where dementia care is also not part of the medical and nursing curricula [20]. Confidence in dementia care for healthcare professionals in Vietnam could be increased by providing training in dementia care, which would also increase exposure to patients with dementia.

Although the Ministry of Health has published a list of medications covered by health insurance, the number of approved medications for the treatment of dementia is limited. Participants were concerned that the type of services and medications available to families were dependent on financial status. A study by Nguyen et al. (2020) suggested that the healthcare policy in Vietnam specifically addressing dementia and other neurological disorders is ineffective and an obstacle to providing quality care [32]. Although one aim of Decision No 155/QD-TTg is to improve dementia care, the current national health insurance program does not provide coverage for expensive pharmacological treatments for dementia and the government-subsidized services [7, 33] are substantially smaller than the cost of the community health services. This is an obstacle to healthcare in China as well, where persons with dementia do not meet the criteria necessary to be eligible for government-funded services offered by community health workers [34].

One way to address the sharp increase in the number of persons living with dementia in Vietnam is to add dementia to the list of chronic diseases. In order to properly address the rapidly ageing population and the increase in the number of persons living with dementia, Vietnam urgently needs to develop a national dementia plan, which will require changing the country's current healthcare system [26]. Vietnam is in the process making an effort to create a national strategy to help address concerns about diagnosis, care, and services for people with dementia and their families in the healthcare system [32].

### Implications

Understanding the viewpoint of healthcare providers in community settings is crucial for planning and executing policies, care support for family members, and educational interventions especially for countries with a growing population of persons living with dementia. This study was conducted to investigate the present care practices offered by community healthcare personnel to older adults living with dementia in Vietnam. The findings demonstrated that the healthcare professionals recognized they lacked training and experience in dementia care. Therefore, it is crucial to initiate and cultivate dementia care education programs aimed at expanding curricula for physicians, physicians’ assistants, and nurses. Including topics relative to dementia in education and ongoing training programs for healthcare professionals could optimize dementia care competencies of community healthcare workers. In addition, the finding that family members of persons with dementia expect, but lack, prompt and continuous care draws attention to the absence of any policy regarding dementia screening or dementia care in the Vietnamese healthcare system. Family members were considered one of the most important components of providing dementia care. Therefore, a means of providing support for family members caring for persons living with dementia must be developed. Finally, the finding that there is lack of attention to early diagnosis of dementia and dementia care indicates formal dementia screening programs must be implemented.

### Limitations

This study had a number of limitations. First, this study was conducted in Ho Chi Minh City, in the south of Vietnam, which is one of the larger cities in Vietnam and might not reflect the practice of healthcare workers in other areas of the country, especially rural areas where access to healthcare for older adults is not always available. Second, in terms of the sample size and the nature of study design, this study was designed as a qualitative study evaluating practice of only 23 community healthcare professionals from ten primary health care facilities, the results might not be generalizable. However, quantitative studies as well as additional qualitative studies are needed to strengthen our findings. Third, although there are guidelines about healthcare for the elderly with dementia, the ‘gold-standard’ model of care at primary care settings is not available especially for developing countries. Therefore, it is not feasible to compare our findings with others. Due to the context-dependent nature of such models, more studies focusing on model of care for the elderly with dementia at grassroot healthcare facilities are also needed. Fourth, the findings of this study might be biased due to the inclusion of physician and physician’s assistants who reported having little training in dementia care. Although their experiences in real life practice at community health facilities provides a broad perspective about patient care, the findings might differ if the participants included only physicians specifically trained in dementia care. Therefore, future studies might consider recruiting physicians from dedicated dementia care facilities.

## Conclusions

This study described the current dementia care provided to older adults by community healthcare professionals in Ho Chi Minh City, Vietnam. This study highlights the need for more educational enrichment for healthcare professionals in Vietnam as well as the need for more research in the field of dementia care in the context of the Vietnamese healthcare system. Findings from this study can serve as real-life evidence to promote the development of community-based policy and care models that could improve the quality of care for patients with dementia in Vietnam. Moreover, this study also reveals an urgent need to have a national dementia strategy, not only to care for those with dementia, but also to support healthcare professionals in this field.

## Data Availability

The datasets generated and/or analyzed during the current study are not publicly available due to the principal investigator’s decision to make the data publicly available upon completion of the formal study. If someone wants to request the data, the first author Hong Le HUYNH-TRUONG can be contacted.

## Abbreviations

ADLs: Activities of Daily Living
GPs: General Practitioners
HCMC: Ho Chi Minh City
LMICs: Low- and Middle-Income Countries
MMSE: Mini Mental State Examination
RACFs: Residential Aged Care Facilities
UMP: University of Medicine and Pharmacy
